# Test-retest reliability of the HEXACO-100—And the value of multiple measurements for assessing reliability

**DOI:** 10.1371/journal.pone.0262465

**Published:** 2022-01-13

**Authors:** Sam Henry, Isabel Thielmann, Tom Booth, René Mõttus

**Affiliations:** 1 Department of Psychology, University of Edinburgh, Edinburgh, Midlothian, United Kingdom; 2 Department of Psychology, University of Koblenz-Landau, Koblenz, Landau, Germany; 3 Institute of Psychology, University of Tartu, Tartu, Estonia; Aalborg University, DENMARK

## Abstract

Despite the widespread use of the HEXACO model as a descriptive taxonomy of personality traits, there remains limited information on the test-retest reliability of its commonly-used inventories. Studies typically report internal consistency estimates, such as alpha or omega, but there are good reasons to believe that these do not accurately assess reliability. We report 13-day test-retest correlations of the 100- and 60-item English HEXACO Personality Inventory-Revised (HEXACO-100 and HEXACO-60) domains, facets, and items. In order to test the validity of test-retest reliability, we then compare these estimates to correlations between self- and informant-reports (i.e., cross-rater agreement), a widely-used validity criterion. Median estimates of test-retest reliability were .88, .81, and .65 (*N* = 416) for domains, facets, and items, respectively. Facets’ and items’ test-retest reliabilities were highly correlated with their cross-rater agreement estimates, whereas internal consistencies were not. Overall, the HEXACO Personality Inventory-Revised demonstrates test-retest reliability similar to other contemporary measures. We recommend that short-term retest reliability should be routinely calculated to assess reliability.

## Introduction

The HEXACO Personality Inventory-Revised (HEXACO-PI-R [1]) is currently one of the most widely used personality questionnaires in psychology and beyond. Several key properties of its domain and facet scales such as convergent and discriminant validity, gender differences, and measurement invariance across various countries and translations have been reported in recent large-sample studies [2, 3]. The scales also demonstrate associations with numerous life outcomes [4–6]. Surprisingly, however, one of the most basic of its psychometric properties, test-retest reliability (or retest reliability; *r*_TT_), has rarely been assessed, and never in sufficiently large samples [7–9].

Reliability denotes “the consistency of a measure with itself” [10; p.464] or of two independent assessments of the same inventory [11]. Whereas internal reliability or internal consistency represents the consistency of responses for a trait using non-identical parallel forms, short-term *r*_TT_ quantifies the extent to which test takers agree with themselves on the *exact same items* being measured at different time points and hence independently, assuming that test takers do not remember their previous responses and copy these at second testing. For many, *r*_TT_ *is* reliability, whereas internal consistency estimates like alpha (α) and omega (ω) are seen to have only been designed to imperfectly approximate reliability when *r*_TT_ is not available [12]. As succinctly noted by McCrae and colleagues [13]: “[i]nternal consistency of scales can be useful as a check on data quality but appears to be of limited utility for evaluating the potential validity of developed scales, and it should not be used as a substitute for retest reliability” (p. 28). In many cases, high internal consistency, reflecting redundancy among test constituents, is even *un*desirable because it may mean poor coverage of the content of the construct being measured–especially when constructs are broad–or an impractically long test. We note that α does not measure internal consistency of items *per se*: with hundreds or thousands of items, any scale has high α but can have practically zero consistency among individual items. Instead, a scale’s α indexes the expected consistency among hypothetical item aggregates containing the same number of items as the scale. Nonetheless, in line with common usage and to clearly distinguish it from *r*_TT_, we will also refer to α as a measure of internal consistency.

In addition to its tighter theoretical adherence to reliability, *r*_TT_ has empirical advantages when compared to internal consistency. Because internal reliability confounds unreliability with unique trait information in items [14], it tends to be lower than *r*_TT_ [15], and this difference may even be underestimated due to occasion-specific state effects inflating internal consistency. Even more importantly, *r*_TT_, but not internal consistency, is a good predictor of scales’ cross-rater agreement (*r*_CA_) and long-term stability, two of the most straightforward and broadly applicable psychometric validity criteria [13, 16]. These associations with validity criteria follow with *r*_TT_ being a more accurate reliability estimate than internal consistency, as reliability necessarily provides an upper bound for validity. Unlike internal reliability, *r*_TT_ can also be estimated for individual items. This provides researchers an empirical criterion to assess item quality–where items with low *r*_TT_ either index characteristics on which respondents cannot even agree with themselves or are simply ambiguous. Although some items may assess a specific pattern of thought, feeling, or behavior that genuinely changes over a brief time span, we contend that traits, even those assessed by single items, should be largely stable over the brief time periods typically used to measure *r*_TT_, and those items with particularly low *r*_TT_s ought to be replaced with more reliable ones where possible.

While indices of internal consistency (most prominently α) have been widely reported for HEXACO measures [2], only a handful of studies have reported *r*_TT_ estimates for them. For example, previous work [9] assessed seven-month *r*_TT_ of the full 200-item HEXACO-PI-R to compare it with a variety of other item properties and found a mean single-item *r*_TT_ = .56 (*N* = 188; *SD =* .10; range = .31 to .80), with mean domain *r*_TT_ = .85 (range = .79 to .90). Others [7] found similar results for seven-month *r*_TT_ of HEXACO domains using the 60-item version of the HEXACO-PI-R (HEXACO-60 [17]), albeit with a very small sample (*N* = 31; *M =* .81; range = .72 to .90). More recently, 2-year *r*_TT_ (*N* = 214) estimates were reported [8] for only the Honesty-Humility, Agreeableness, and Conscientiousness domains and facets of the 100-item HEXACO-PI-R (HEXACO-100 [18]). Domain reliabilities were *r*_TT_ *=* .80, .75, and .74, respectively, and median facet reliability was *r*_TT_ = .69 (range = .58 to .78).

Despite providing good preliminary evidence for the temporal stability of HEXACO domains, all three studies used a testing interval more appropriate for long-term stability estimates, rather than for quantifying *r*_TT_ [19]. Longer retest intervals over many months or even years confound unreliability (or “transient error”) with actual personality change, while short-term intervals such as two weeks [20, 21] or less [22] are more appropriate to quantify *r*_TT_ because true change is unlikely to occur, but test takers are also unlikely to retrieve their previous responses from memory. Cattell et al. [23] recommended one or two months as the ideal interval where no true change should occur and “memory effects” [24] are still unlikely. However, more recent research [20] suggests that even shorter timespans still do not evoke memory effects, finding no discernible difference in Big Five (i.e., NEO) personality domain *r*_TT_s measured two months versus two weeks apart. For single items, Kosinski et al. [25] found that median *r*_TT_s declined almost linearly from one-day to one-, two-, three-, and four-week retest intervals, though the range was relatively narrow (*r*_TT_s = .63 to .70) and did not include a two-month interval for further comparison. Thus, while the exact interval that distinguishes between unreliability and true change is unclear (and perhaps impossible to resolve), we suggest–in line with previous research [e.g., 16, 20, 26]–that two weeks strikes a balance between mitigating both the possibility of true change and the likelihood that participants recall and repeat their responses from the first measurement.

Given the popularity of HEXACO-PI-R scales and that *r*_TT_ is likely a superior measure of the scales’ most fundamental property, reliability, a comprehensive study of HEXACO-PI-R domain, facet, and item *r*_TT_s over a brief time period should be of wide interest to both personality researchers and those applying HEXACO-PI-R scales in other (including practical) settings. Thus, we report those estimates here for the English HEXACO-100 and HEXACO-60 (where the latter uses a subset of items from the former); although the longer 200-item version exists, the HEXACO-100 and HEXACO-60 are much more commonly used (in a meta-analysis [2], the two shorter scales were used in 443 of 489 studies that administered a HEXACO-PI-R inventory). To evaluate the criterion validity of *r*_TT_ versus internal consistency estimates, we also explore the extent to which these indices correlate with facet-scale and (where possible) single-item *r*_CA_s [18]. Thus, similarly to previous work [13], we expected *r*_TT_ but not internal consistency estimates to correlate with *r*_CA_s at all levels of investigation. Finally, we report the *r*_TT_s for individual HEXACO-PI-R items, which could suggest those in need of replacement in future iterations of the inventory and inform research on the properties of (un)reliable items.

## Material and methods

### Participants

Participants were recruited via Prolific Academic from a cohort of participants (*N* = 639) that forms part of an ongoing project led by the first and last author on this manuscript. These participants had previously provided survey responses for personality, life outcomes, and item properties. All participants provided informed, written consent online in all previous waves of data collection, which was approved by the University of Edinburgh School of Philosophy, Psychology, and Language Sciences Research Ethics Committee (Refs 123-1920/1 and 123-1920/2). The present study, submitted as a new iteration of the previous one, received approval from the same committee on 11 September 2020 (Ref 400-1920/3).

We invited these participants to complete the English HEXACO-100 [18] twice in two weeks. At the first administration (T1), participants gave written online consent before being directed to the survey. T1 was released at 11:55 GMT on 23 September 2020 and closed at 19:01 GMT on 28 September after achieving the planned sample size (*N* = 450). The second survey (T2) was released in two waves to account for variance in T1 start dates and restrict the range of retest intervals. Specifically, we published the first T2 survey at 11:15 GMT on 6 October for participants who completed T1 on 23–25 September (*n* = 421), then published the second wave two days later (12:11 GMT on 8 October) for participants who completed T1 between 26 and 28 September.Ultimately, 423 participants (48.5% female; *M* age = 26.9, *SD* age = 7.9, age range = 19 to 69) completed both T1 and T2 assessments. Pending a successful quality control check, all participants received a total compensation of £2.00 for participation at both time points.

Following recommendations from a similar study [27], we excluded participants whose profile consistency (*q*, calculated as the overall correlation between responses across all items at each measurement occasion) was exceptionally low–they used a cut-off of *q* = 0.25 for consistency estimates taken from repeated measures in the same session. Given our considerably longer testing interval, we were more lenient and only removed participants whose profile consistency was three or more standard deviations below the median *q* = 0.66 (i.e., *q* ≤ 0.12). This excluded seven participants, leaving a final sample of *N* = 416 (49.0% female; *M* age = 26.9, *SD* age = 7.9, age range = 19 to 69). For a hypothesized mean *r*_TT_ of .65 (based on previous research [16, 25]), this final sample size entailed an average predicted standard error (*SE*) of .037.

With respect to other demographic variables, our sample was rather heterogeneous. No one country of birth exceeded 100 participants, with the largest representation coming from Portugal (*n* = 84), United Kingdom (*n* = 76), Poland (*n* = 49), Italy (*n* = 33), Greece (*n* = 30), Spain (*n* = 22), and the United States (*n* = 15). All other nations had *n*s < 10. English was underrepresented as a first language, with just under a quarter of the sample being native English speakers (*n* = 94); first language mapped fairly consistently with country of birth, although there was a greater variety of the latter (50 countries of birth versus 33 first languages).

Finally, about 1/3 (*n* = 137 and 135, respectively) of the sample was missing data for student and employment status, but among the remaining subsamples, about half (53.1%) were students and over two thirds (69.4%) claimed to be employed in some capacity. In summary, although our sample was not representative of any one population, we did manage to capture a relatively wide variety of cultures, ages, and occupational circumstances.

### Measure

The HEXACO-100 measures the six HEXACO domains, their respective four facets (see Table 1 for domain and facet names), and an additional interstitial facet—Altruism—for a total of 25 facets with four items each [18]. The measure also contains all 60 items of the shorter form of the inventory, the HEXACO-60 [17], which contains 10 items for each domain. The HEXACO-60 does not include items for the Altruism facet, and is generally not intended to assess HEXACO facets. Full scales and scoring keys are freely available at https://hexaco.org/hexaco-inventory.

**Table 1:** Empirical properties of HEXACO-100 domains and facets.

Scale	Our study			Lee & Ashton (2018)		
	α	*r*_TT_	Δ	*r*_CA_	α_Student_	α_Online_
H: Honesty-Humility	.88	.89	.01	.46	.82	.89
E: Emotionality	.84	.88	.04	.61	.84	.84
X: eXtraversion	.89	.92	.03	.56	.85	.86
A: Agreeableness	.87	.86	(.01)	.47	.84	.86
C: Conscientiousness	.87	.88	.01	.52	.84	.82
O: Openness to Experience	.83	.88	.05	.56	.81	.82
H1: Sincerity	.83	.75	(.08)	.20	.66	.78
H2: Fairness	.85	.86	.01	.45	.76	.83
H3: Greed Avoidance	.83	.84	.01	.47	.81	.83
H4: Modesty	.79	.80	.01	.30	.68	.79
E1: Fearfulness	.72	.81	.09	.51	.70	.70
E2: Anxiety	.75	.81	.06	.40	.64	.73
E3: Dependence	.78	.80	.02	.44	.80	.76
E4: Sentimentality	.78	.84	.06	.47	.70	.73
X1: Social Self-Esteem	.74	.85	.11	.38	.67	.70
X2: Social Boldness	.74	.83	.09	.53	.76	.72
X3: Sociability	.80	.85	.05	.45	.71	.77
X4: Liveliness	.81	.83	.02	.45	.76	.78
A1: Forgivingness	.82	.78	(.04)	.35	.74	.78
A2: Gentleness	.73	.75	.02	.35	.66	.72
A3: Flexibility	.70	.76	.06	.35	.61	.64
A4: Patience	.82	.82	0	.43	.79	.80
C1: Organization	.74	.81	.07	.52	.74	.73
C2: Diligence	.80	.83	.03	.37	.70	.71
C3: Perfectionism	.76	.79	.03	.42	.69	.69
C4: Prudence	.77	.74	(.03)	.33	.69	.70
O1: Aesthetic Appreciation	.69	.83	.14	.49	.66	.65
O2: Inquisitiveness	.66	.80	.14	.45	.66	.70
O3: Creativity	.78	.87	.09	.50	.75	.73
O4: Unconventionality	.54	.78	.24	.36	.52	.59
Interstitial: Altruism	.66	.75	.09	.36	.59	.66
*Domain Median*	.*87*	.*88*	.*01*	.*54*	.*84*	.*85*
*Facet Median*	.*77*	.*81*	.*04*	.*43*	.*70*	.*73*

α = alpha internal consistency. *r*_TT_ = 13-day test-retest reliability. *r*_CA_ = cross-rater agreement. Δ = *r*TT—α in the present sample, with instances of α > *r*_TT_ in parentheses.

### Analyses

After recoding reverse-keyed items, we calculated domain and facet scale scores for both HEXACO-100 and HEXACO-60 using mean scores of their associated items. Though facet scores are not typically calculated for the latter, we deemed it worthwhile to do so for the purpose of comparing αs and *r*_TT_s. Using the psych() package in R Version 4.1.1 [28, 29], we calculated internal consistencies (Cronbach’s α) for facets and domains (using item scores to estimate domains’ αs). Because some researchers [12] recommend omega (ω) as a more appropriate measure for internal reliability than α, we calculated domain and facet ωs for comparison with αs. They correlated .98 and .99, respectively; ω estimates are available in the Online Supplement (https://osf.io/wz3du/). Test-retest reliabilities for domains, facets, and items were all estimated as the correlation between their scores at T1 and T2.

We report α and *r*_TT_ estimates from our sample alongside the α and *r*_CA_ estimates from a previous meta-analysis [18]. They reported the estimates from a large sample comprised of self- and informant reports from university students (*N* = 2,863 pairs), and a very large sample of participants providing only self-reports via a survey link on the HEXACO website (*N* = 100,318). Full details on the samples, data collection, and results may be found in the original publication [18].

Finally, we report Spearman’s correlations between αs, *r*_TT_s, and *r*_CA_s of HEXACO-100 facets as well as the correlation between items’ *r*_TT_s and *r*_CA_s. Items’ *r*_CA_s were not published in the original paper, but the authors kindly made their data available to us, so we calculated these. We opted not to conduct these analyses for domains because we deemed that correlations of vectors of six values would not provide additional informative value. We were particularly interested in the extent to which facets’ *r*_TT_ and α correlated with *r*_CA_: in other words, how strongly reliability criteria correlate with a common validity criterion.

Code and data that may be used to reproduce all analyses, as well as a data file containing final outputs, can be found in the Online Supplement at https://osf.io/wz3du/.

## Results

### Completion time, interval length, and language

Median time to complete the survey at T1 was 11’ 33”, (SD = 8’22”, IQR = 8’55” to 16’3”). For T2, we removed times for two participants with extreme values (approximately 25 and 72 hours). The resulting completion times were slightly shorter than T1 (median = 10’44”, SD = 9’20”, IQR = 8’13” to 13’ 20”). On average, participants provided complete data in about 13 days (median T1-T2 interval = 12 days, 23 hours, 37 minutes; *SD* = 1 day, 6 hours, 41 minutes), with a vast majority completing the survey by the end of two weeks; only *n* = 56 completed the survey after 14 days and no participants took more than 19 days to complete it.

To evaluate whether interval length moderated *r*_TT_ estimates, we calculated mean T1-T2 profile correlations for each participant, then correlated these with the length of time in seconds between T1 and T2 start times. The resulting correlation was *r* = .07 (*p* = .14), suggesting no relationship between the number of days between administrations and overall reliability. We also examined correlations between T1-T2 profile correlations and time taken to complete the survey at T1, T2, and the average of these, but found no evidence that delay between measurements affected participants’ overall consistency over time either: *r*s = -.08 (*p* = .12), .01 (*p* = .84), and -.04 (*p* = .42), respectively.

Finally, given that most participants were non-native English speakers, we considered it important to test for potential differences in profile correlations between T1 and T2 assessments. Indeed, natives showed slightly higher average T1-T2 profile correlations (*q* = .69) than non-natives (*q* = .62). By implication, the *r*_TT_ estimates provided here may thus underestimate reliability, providing a particularly conservative test. These estimates are slightly lower than the minimum recommendation for profile correlations of .70 based on a same-day retest interval [27], which is expected given our longer retest interval.

### HEXACO-100 domain and facet scales

As summarized in Table 1, domains had a median 13-day *r*_TT_ of .88 (*SD* = .02, range = .86 to .92) versus a median α of .87 (*SD* = .02, range = .83 to .89). These αs, slightly lower than *r*_TT_s for all but one domain (i.e., Agreeableness), were comparable to those from the comparison study by Lee and Ashton ([18]; *Mdn* = .88, *SD* = .03, range = .82 to .89) that we report in Table 1, as well as a more recent meta-analysis ([2]; *Mdn =* .84, *SD* = .02, range = .81 to .86). For facets, median *r*_TT_ and α were .81 (*SD* = .04, range = .74 to .87) and .77 (*SD* = .07, range = .54 to .85), respectively. Our αs tended to be higher than those observed by Lee and Ashton ([18]; *Mdn*s = .70 and .73), although our rankings correlated highly with their student and online samples (ρs = .67 and .88; Table 2). In turn, facet *r*_TT_s were higher than αs for all but three facets (mean difference *r*_TT_−α: Δ = .04, median = .03, range = -.08 to .24). Though typically small, the disparities were more notable for facets with lower αs, as evidenced by a correlation of *ρ* = -.72 between α and Δ. For example, in the lowest quintile of αs (range = .54 to .70), the median difference between α and *r*_TT_ was .14 (range = .06 to .24), whereas disparities were negligible in the highest quintile (α range = .82 to .85; Δ median = .0, range = -.08 to .01).

**Table 2:** Spearman correlations of *r*_TT_, α, and *r*_CA_ of HEXACO facets.

Estimate	α	*r*_TT_	*r*_CA_	LA α_Student_
*r*_TT_	.34			
*r*_CA_	‒.07	.69		
LA α_Student_	.67	.55	.52	
LA α_Online_	.88	.36	.07	.69

α = alpha internal consistency estimates from our sample, averaged across both testing occasions. *r*_TT_ = 13-day retest reliability. *r*_CA_ = cross-rater agreement assessed by Lee & Ashton (2018) [18]. LA refers to the two samples in which Lee & Ashton (2018) calculated α, with appropriate subscripts.

Details on inter-item correlations for domains, facets, and all items can be found in the Online Supplement.

### Relationships between r_TT_, α, and the validity criterion r_CA_ for HEXACO-100 facets

Table 2 reports Spearman correlations between *r*_TT_s, αs, and *r*_CA_s for the HEXACO-100 facets. While *r*_CA_s correlated strongly (*ρ* = .52) with only one of the three α values–α_Student_, from the same sample–they demonstrated a stronger association with the *r*_TT_ values from the present sample (*ρ* = .69). Because *r*_TT_ was also correlated with α_Student_ (ρ = .55), we conducted a *post-hoc* analysis to determine the partial associations among these three properties. When controlling for *r*_TT_, the relationship between α_Student_ and *r*_CA_ was substantially attenuated (*ρ* = .24). However, correcting the correlation between *r*_TT_ and *r*_CA_ for α_Student_ only reduced the association to *ρ* = .56.

### HEXACO-100 Items

Median 13-day *r*_TT_ of the HEXACO-100 items was .65 (*M* = .65, *SD* = .08, range = .39 to .84). The average standard error of the correlations was .037 (range = .027 to .045). Estimates of *r*_TT_s, standard deviations, and standard errors for all items of the HEXACO-100 can be found in S1 Table, with the five highest and lowest available in Table 3. Similar to the relationship found at the facet level, single-item *r*_TT_ estimates correlated *ρ* = .62 with their *r*_CA_s. Interestingly, a *post-hoc* analysis found that item *r*_TT_ and *r*_CA_ were also highly correlated with the items’ standard deviation: *ρ*s = .74 and .58, respectively.

**Table 3:** HEXACO-100 items with highest and lowest test-retest reliabilities.

Item	Code	*r*_TT_
**Low**		
*I wouldn’t pretend to like someone just to get that person to do favors for me*	H1	0.39
*I wouldn’t want people to treat me as though I were superior to them* *	H4	0.46
*I don’t allow my impulses to govern my behavior* *	C4	0.47
*I generally accept people’s faults without complaining about them* *	A2	0.48
*I wouldn’t use flattery to get a raise or promotion at work*, *even if I thought it would succeed*	H1	0.48
**High**		
*I find it boring to discuss philosophy*	O4	0.84
*If I had the opportunity*, *I would like to attend a classical music concert*	O1	0.83
*I would be quite bored by a visit to an art gallery*	O1	0.80
*If I knew that I could never get caught*, *I would be willing to steal a million dollars* (R)	H2	0.79
*I sometimes feel that I am a worthless person* (R)	X1	0.77

Codes correspond to facet labels given in Table 1. *r*_TT_ = 13-day test-retest reliability.

* indicates items not included in the HEXACO-60. (R) = reverse-keyed.

### HEXACO-60

Properties of the HEXACO-60 demonstrated similar patterns as the HEXACO-100, with *r*_TT_ being slightly higher than α on average. Domains had a median *r*_TT_ = .86 (*SD* = .02, range = .82 to .89) and median α = .82 (*SD* = .03, range = .80 to .87), with all α*s ≤ r*_TT_. Median facet *r*_TT_ was .76 (*SD* = .05, range = .68 to .87) and median α was .72 (*SD* = .08, range = .52 to .84). We again found the association between *r*_CA_ and *r*_TT_ for facets, *ρ* = .57. This held for single items as well (*ρ =* .67), for which the median 13-day *r*_TT_ was .65 (*SD* = .09, range = .39 to .84). As noted in Table 3, while three of the five lowest *r*_TT_ items from the HEXACO-100 do not feature in the HEXACO-60, all five of the items with highest *r*_TT_ are included in the shorter scale. A full breakdown of properties for HEXACO-60 domains, facets, and items can be found in the Online Supplement.

## Discussion

The HEXACO-PI-R, and particularly its two shorter versions, the HEXACO-100 and HEXACO-60, are currently among the most widely-used personality questionnaires. Surprisingly, however, the test-retest reliability (*r*_TT_)–a key psychometric property of any psychological test–of their scales and items has not yet been systematically studied. Although several studies have reported HEXACO-PI-R stability estimates over intervals from a few months up to two years [7–9], we herein provided the first examination of *short-term r*_TT_ for the English HEXACO-100 and HEXACO-60 to our knowledge. We found that *r*_TT_s were slightly higher on average on both the domain and facet levels than internal consistencies (α), suggesting that the latter can underestimate scales’ reliabilities, particularly at the lower end. *r*_TT_s remained high even for those facets with low αs, which indicates that their items are likely reliable, just less intercorrelated than those of scales with a higher α. We also found evidence to suggest that α–but not *r*_TT_−confounds reliability with non-random error sources of variance, indicated by strong associations between *r*_TT_ and cross-rater agreement (*r*_CA_), a common validity criterion, even when controlling for α.

The present study indicates that HEXACO-PI-R scales have comparable reliability as other established personality measures (see below). This is good news for researchers and practitioners, as the HEXACO-PI-R scales are freely available, meaning these measures can be applied without any costs that are associated with other proprietary personality measures. Moreover, our finding that the HEXACO-60 assesses HEXACO-PI-R domains, facets, and items with similar degrees of reliability to its longer 100-item variant supports its use in settings where a shorter version may be favored, such as clinical use or inclusion in a larger research project assessing many other variables. The finding that facet properties in the HEXACO-60 were consistent with the longer HEXACO-100, despite its being written only to measure domains, may suggest that researchers could consider interpreting facets when measuring the HEXACO domains with the shorter version, although this would need to be confirmed by predictive validity studies. In sum, our findings contribute further empirical backing for the use of HEXACO scales in research and practice.

### Striking similarities to other measures

The HEXACO-PI-R *r*_TT_s observed here were remarkably similar to those reported for other popular personality scales, such as the Big Five, at all levels of the trait hierarchy. For example, McCrae and colleagues [13] found that median NEO-Personality Inventory-Revised (NEO-PI-R [30]) αs ranged from .71 to .77 in three samples, with an overall range of .50 to .87—compared to .54 to .85 in the present study. NEO-PI-R facet *r*_TT_s also showed highly similar patterns to the present study, with median *r*_TT_ = .82 and a slightly wider range of *r*_TT_s = .72 to .89 (where median facet *r*_TT_ of the HEXACO-100 in our study was = .81, range = .74 to .87). McCrae and colleagues found consistent associations between *r*_CA_ and *r*_TT_, but none with αs after controlling for third variables, just as we observed in the present study.

Single items assessing the Big Five domains and facets also demonstrated similar levels of *r*_TT_ to the present study. A recent study [16] reported median *r*_TT_ of .64 *(M* = .64, *SD* = .09, range = .36 to .87) for the NEO-PI-R, whereas other research found median *r*_TT_ of .66 (data for the *SD* and range were not available) for a 100-item IPIP scale [21, 25, 31]. The HEXACO-PI-R showed a similar *r*_TT_ range as the NEO-PI-R, although the former was slightly narrower (i.e., *r* = .39 to .84 vs .36 to .87, respectively). The association between *r*_CA_ and *r*_TT_ has also been found for NEO-PI-R items (*ρ* = .57, *p* < .001 [16]). These results suggest three things: (1) ever-accumulating evidence indicates that the “average” personality item is reliable at about *r*_TT_ = .65; (2) items within contemporary scales vary substantially in quality, as indicated by several especially unreliable items; and (3) *r*_TT_ is a good predictor of items’ validity, making it useful quality criterion in scale development.

### Variation in item properties: Interpretations and implications

An inventory’s reliability is a fundamentally desirable psychometric property, and a guiding theoretical claim of this manuscript has been that *retest* reliability in particular is a better index of reliability than other indices, particularly the more commonly-used Cronbach’s α. We have also shown this empirically to be the case, with *r*_TT_s > αs on average, and *r*_TT_ consistently linked with validity criteria whereas α is not. But *why* should this be the case?

McCrae [14] offers one explanation, describing how to use the *r*_TT_, α, and *r*_CA_ of a given trait to parse its variance into common trait, method, and (items’) specific variance components. In essence, the model postulates: Most items have unique valid variance [21, 32], and this unique variance is by definition *not* captured by α but *is* assessed by *r*_TT_ (because α removes anything not common to all items); therefore, a trait scale that aggregates multiple items should have *r*_TT_ > α. Our results support this model, with only three facet αs lower than *r*_TT_s. In other words, most facet measures contain both information that is common to items written to measure the trait (e.g., Sincerity) and unique valid content specific to each item, ostensibly indexing a further personality *nuance* [14]. However, the ways that items vary in their individual *r*_TT_s and contributions to higher-order trait αs is as of yet a relatively unexplored question.

#### The role of item content

As one starting point, we can compare how domains and facets in the HEXACO-100 differ in their *r*_TT_s and αs. Of the top 10 most reliable items, Openness to Experience and Extraversion featured with three and four items, respectively. Conversely, Honesty-Humility and Agreeableness demonstrated the opposite pattern, containing three and four of the *least* reliable items. An examination of the least reliable items suggests that they may be especially dependent on the *contextuality* of the item, particularly when it describes a hypothetical “other.” For example, responses to the least reliable item “I wouldn’t pretend to like *someone* just to get that person to do favors for me” (emphasis added) may depend on a variety of circumstantial details the respondent imagines when responding: who is the “someone” and what relation do they have to me? Are they a friend, colleague, boss, or stranger? How important is the favor? The same participant could envision different situations with different individuals, stakes, and emotional investment when responding to the item on different testing occasions, ultimately leading to a low *r*_TT_ on average. At face value, other low-*r*_TT_ items seem to demonstrate similar levels of ambiguity (e.g., “I generally accept *people’s* faults without complaining about them,” “I wouldn’t want *people* to treat me as though I were superior to them”; emphasis added) that leave the context unclear.

In contrast, the *most* reliable items are generally less vague in their contextual referents. “I find it boring to discuss philosophy,” “If I had the opportunity, I would like to attend a classical music concert,” and “I would be quite bored by a visit to an art gallery” are the three items with highest *r*_TT_. While all three items assess Openness to Experience, more notable is that the content references specific situations that do not evoke the presence of a particular other individual. These Openness to Experience items leave little to the respondent’s imagination.

Conversely, facets of Openness to Experience had the *lowest* α on average, with α for Unconventionality, Inquisitiveness, and Aesthetic Appreciation all falling below .70. They also had the greatest disparities between α and *r*_TT_, with the latter at or near the median, and respective differences of .24, .14, and .14. This demonstrates two things. More generally, it serves as a reminder that a scale’s actual reliability (as assessed by *r*_TT_) is not well approximated by α. With regard to the Openness to Experience facets in particular, it may well be that the facet-level traits assessed are more abstract and therefore broader in content than facets of other domains such as, for example, Greed Avoidance from the Honesty-Humility domain–which has, in turn, one of the highest α values.

These speculations can and should be tested empirically. One way to explore questions of item content more generally is by recruiting a small pool of lay or expert raters (say, *N* = 20–30 [33]) to assess the degree to which items differentially feature properties (including contextuality) that may be relevant for a variety of empirical criteria, such as *r*_TT_ or *r*_CA_. This would shed light on how the way items are written or the trait they are assessing is related to their empirical properties. Item ratings could also be averaged to generate scores for the broader facets they measure, which could then be compared to a rating of the same criterion but for the facet (or domain) alone. For example, how would a breadth rating for “Unconventionality” as a facet compare to the average of breadth ratings across its constituent items? Investigation of the nuanced ways in which measures of personality vary in content and at different levels of specificity could help generate new questions and hypotheses in future scale development.

Some work on these types of questions has already begun. Previous research [9] found that item variance strongly predicted items’ *r*_CA_ for both the NEO-PI-3 [34] and the full HEXACO-PI-R, as well as *r*_TT_ for the latter, but not single-item internal reliability. *r*_TT_ and *r*_CA_ were also associated with item evaluativeness, observability, position, length, inclusion of a negation, and broad content domain (assigning Big Five and HEXACO domains to either “Engagement” or “Altruism”), although these factor loadings were more modest [9].

These are useful preliminary findings, but more properties still may be investigated to explore as of yet unexplained mechanisms driving items’ validity. For example, the item properties studied by De Vries and colleagues [9] only achieved *R*^*2*^ values of .06 and .17 for item variance, the strongest predictor of *r*_TT_ and *r*_CA_. Perhaps studying some of the aforementioned properties such as breadth, contextuality, and abstractness may clarify this issue; others have suggested additional candidates such as item ambiguity, complexity, and (social) importance [33]. Varying domains of content, such as the affective, behavioral, cognitive, and motivational components of items have also been suggested as possible candidates and thus preliminarily studied [35].

We would thus call for an integration of and extension to these varied lines of research. For example, while De Vries and colleagues [9] assessed *r*_TT_ using data more appropriate for long-term stability estimates, follow-up work using the present findings could refine the relationships between *r*_TT_ and item properties. Likewise, researchers could attempt to incorporate analyses of item content into studies that apply the variance decomposition techniques suggested by McCrae [14]. By drawing on the insights and resources from past work and across tests, we can iteratively make progress toward a comprehensive understanding of how items behave and why.

#### Implications for scale development

Taken together, the findings in the present study and subsequent examination of item properties may also offer personality researchers the potential to revise existing scales in an informed way, with the aim of keeping only those items that best measure the target traits. This, in turn, could result in survey administrations that capture more and better information with the same or even a smaller number of items, helping researchers to maximize the investment of their resources while also having more confidence in observed relationships with outcome variables of interest.

How exactly to assess the “goodness” of an item remains an open question, as evidenced by the previous section, but we would recommend that researchers begin by prioritizing items with higher *r*_TT_s, standard deviations, and *r*_CA_s, three highly intercorrelated empirical properties [9, 21, the present study]. We caution, though, that comparisons of these properties should only be used when choosing between items *for a single trait*, as it is conceivable that some traits may have reliability “ceilings” such that they are more difficult to assess than others–both for an individual about themselves or for an informant who knows them well–but this does not necessarily make them less of a trait. Further, the interpretation of these criteria is not always clear-cut; indeed, at least two criteria are logically intertwined: specifically, the stability of one’s self-view (*r*_TT_) is probably a necessary condition for an informant to agree with them (*r*_CA_). Given the complexities of interpreting even apparently straightforward quality indices of items, let alone the complex interplay of factors at both the level of the written item and regarding the nature of the underlying trait itself, we are currently hesitant to offer concrete advice for survey generation. Instead, we encourage researchers to pursue questions–including those we pose above–that begin to paint a clearer picture of how items, traits, and their properties interact.

### Limitations and generalizability

A limitation of the present study is that we have only estimated *r*_TT_ for the HEXACO-100, meaning half of the items that assess the HEXACO domains (as per the full HEXACO-PI-R) remain untested. Furthermore, both this study and much of the research that we have cited on *r*_TT_ reports on relatively small samples. Measures for what arguably are the two most popular models of personality (HEXACO and Big Five) have *r*_TT_ estimates based on samples *N* < 500, which rather pale in comparison to cross-sectional samples of (hundreds of) thousands of self- and informant-reports. This likewise limits the ability to generalize our findings, particularly given 1) the disproportionate number of non-native English speakers, and their lower overall consistency in responses compared to natives, which may have downwardly-biased our reliability estimates; and 2) that the sample was recruited using a paid online service, perhaps leading to a selection bias. To the former point, this may actually serve as encouraging, suggesting that the *r*_TT_s reported here are actually lower limits for HEXACO-PI-R domain, facet, and item *r*_TT_. To the latter, the αs observed in the present study are consistent with the substantially larger samples in recent studies and meta-analyses [2, 18], suggesting that our data were not irregular.

Finally, though we chose an approximately two-week retest interval primarily for empirical consistency with previous work, we found little robust theoretical rationale in the personality literature as to why to prefer two weeks to, say, one, three, four, six, or eight–aside from vague assumptions about true trait change and memory effects. As study of single-item properties in particular advances, researchers should investigate more thoroughly the differences in reliability across different retest intervals, while also integrating findings from the memory literature, to inform our understanding of appropriate retest intervals.

## Conclusions

Given (1) facet α estimates being generally lower than *r*_TT_, (2) the unique, robust association between *r*_TT_s and *r*_CA_s, and (3) the remarkable replicability of these findings across multiple samples, questionnaires, and even models of personality, we reiterate the growing sentiment that *r*_TT_ is a superior estimate of scale reliability to α (or its cousin ω). This is not a new position [11, 36], but it is one that has been overlooked given the convenience of estimating internal consistency. However, as participants become more readily available via online recruitment platforms, we see little reason to avoid–and much to be gained by–collecting *r*_TT_ data as a standard procedure in scale development. We thus recommend calculation of *r*_TT_ as routine practice in psychological research and argue that internal consistency should only be used to screen for data quality [13].

We conclude by advising researchers to pay special attention to item content when designing scales. The average personality item appears to be reliable at approximately *r*_TT_ = .65. However, as shown in various samples, many items are still quite unreliable, ranging as low as the .30s. We suggest that the wide variability in *r*_TT_ across personality scales is, in part, due to the inclusion of “poor” items, rather than solely indexing true variation in reliability across traits. We argue that *r*_TT_ provides one straightforward means of identifying lower-quality items and replacing them with higher-quality ones [33, 37]. We also call for further investigation into the properties that are predictive of *r*_TT_ and related validity criteria (e.g., *r*_CA_, heritability, long-term stability). Ultimately, we encourage researchers to deliberately explore how components of item *content* relate to item *quality*, while at the same time considering the traits they intend to measure. We believe that devoting time and resources to these sorts of questions will move personality measurement–and thus our entire field–forward.

## Supporting information

S1 Table HEXACO-100 items and their descriptive statistics. *r*_TT_ = 13-day test-retest reliability. *SE* = standard error for the *r*_TT_ estimate. *SD* = Standard deviation of the item. * indicates items not included in the HEXACO-60.
(DOCX)

S1 File Raw data for HEXACO-100 items, facets, and domains. (XLSX)

S2 File Facet and factor omegas for HEXACO-100 and HEXACO-60. (XLSX)
