# Supplementary material for: Test-retest reliability of the HEXACO-100—And the value of multiple measurements for assessing reliability
Source: PLoS One. 2022 Jan 13;17(1):e0262465. doi: 10.1371/journal.pone.0262465 (PMC8757920; doi:10.1371/journal.pone.0262465)
Supplement: S1 Table — rTT = 13-day test-retest reliability. SE = standard error for the rTT estimate. SD = Standard deviation of the item. * indicates items not included in the HEXACO-60. (DOCX) [file pone.0262465.s001.docx]

| S1 Table. HEXACO-100 items and their descriptive statistics. |  |  |  |  |
| --- | --- | --- | --- | --- |
| Item | *r*_TT_ | *SE* | *SD* | Facet Code |
| I find it boring to discuss philosophy | .84 | .027 | 1.03 | O4: Unconventionality |
| If I had the opportunity, I would like to attend a classical music concert | .83 | .028 | 1 | O1: Aesthetic Appreciation |
| I would be quite bored by a visit to an art gallery | .8 | .029 | 1 | O1: Aesthetic Appreciation |
| If I knew that I could never get caught, I would be willing to steal a million dollars | .79 | .03 | .86 | H2: Fairness |
| I sometimes feel that I am a worthless person | .77 | .031 | .72 | X1: Social Self-Esteem |
| In social situations, I'm usually the one who makes the first move | .76 | .032 | .98 | X2: Social Boldness |
| People often call me a perfectionist | .76 | .032 | .76 | C3: Perfectionism |
| I clean my office or home quite frequently* | .75 | .033 | .98 | C1: Organization |
| I feel like crying when I see other people crying | .75 | .033 | .96 | E4: Sentimentality |
| I feel reasonably satisfied with myself overall | .75 | .033 | 1.01 | X1: Social Self-Esteem |
| I prefer jobs that involve active social interaction to those that involve working alone | .75 | .033 | 1 | X3: Sociability |
| I dont think of myself as the artistic or creative type | .74 | .033 | .84 | O3: Creativity |
| I want people to know that I am an important person of high status | .74 | .033 | .71 | H4: Modesty |
| I would like to be seen driving around in a very expensive car* | .74 | .033 | 1.03 | H3: Greed Avoidance |
| On most days, I feel cheerful and optimistic | .74 | .033 | .91 | X4: Liveliness |
| People think of me as someone who has a quick temper | .74 | .033 | 1.09 | A4: Patience |
| I would enjoy creating a work of art, such as a novel, a song, or a painting | .73 | .034 | 1.05 | O3: Creativity |
| I'd be tempted to use counterfeit money, if I were sure I could get away with it | .73 | .034 | .96 | H2: Fairness |
| People often joke with me about the messiness of my room or desk* | .73 | .034 | 1.22 | C1: Organization |
| I tend to feel quite self-conscious when speaking in front of a group of people* | .72 | .034 | .98 | X2: Social Boldness |
| I would like to live in a very expensive, high-class neighborhood | .72 | .034 | .95 | H3: Greed Avoidance |
| I avoid making "small talk" with people* | .71 | .035 | .88 | X3: Sociability |
| I feel that I am an unpopular person | .71 | .035 | .91 | X1: Social Self-Esteem |
| People have often told me that I have a good imagination | .71 | .034 | 1.04 | O3: Creativity |
| I do only the minimum amount of work needed to get by | .7 | .035 | .85 | C2: Diligence |
| I enjoy looking at maps of different places* | .7 | .035 | 1.09 | O2: Inquisitiveness |
| I would like a job that requires following a routine rather than being creative* | .7 | .035 | 1.17 | O3: Creativity |
| I would never accept a bribe, even if it were very large | .7 | .035 | .73 | H2: Fairness |
| If I want something from someone, I will laugh at that persons worst jokes* | .7 | .035 | .83 | H1: Sincerity |
| When someone I know well is unhappy, I can almost feel that persons pain myself* | .7 | .035 | 1.01 | E4: Sentimentality |
| Even in an emergency I wouldn't feel like panicking | .69 | .035 | 1.16 | E1: Fearfulness |
| I am energetic nearly all the time* | .69 | .035 | .96 | X4: Liveliness |
| I don't mind doing jobs that involve dangerous work* | .69 | .036 | .99 | E1: Fearfulness |
| I rarely discuss my problems with other people* | .69 | .035 | 1.08 | E3: Dependence |
| Often when I set a goal, I end up quitting without having reached it* | .69 | .035 | .99 | C2: Diligence |
| When working, I often set ambitious goals for myself* | .69 | .036 | .83 | C2: Diligence |
| I remain unemotional even in situations where most people get very sentimental | .68 | .036 | .94 | E4: Sentimentality |
| I would get a lot of pleasure from owning expensive luxury goods | .68 | .036 | .97 | H3: Greed Avoidance |
| People sometimes tell me that I'm too stubborn | .68 | .036 | .95 | A3: Flexibility |
| When I'm in a group of people, I'm often the one who speaks on behalf of the group | .68 | .036 | 1.04 | X2: Social Boldness |
| I enjoy having lots of people around to talk with* | .66 | .037 | 1.02 | X3: Sociability |
| I think of myself as a somewhat eccentric person* | .66 | .037 | .99 | O4: Unconventionality |
| I'm interested in learning about the history and politics of other countries | .66 | .037 | .85 | O2: Inquisitiveness |
| My attitude toward people who have treated me badly is "forgive and forget" | .66 | .037 | 1.01 | A1: Forgivingness |
| People often tell me that I should try to cheer up* | .66 | .037 | 1.22 | X4: Liveliness |
| People sometimes tell me that I am too critical of others | .66 | .037 | .83 | A2: Gentleness |
| I worry a lot less than most people do | .65 | .037 | 1.02 | E2: Anxiety |
| I would be tempted to buy stolen property if I were financially tight* | .65 | .037 | .96 | H2: Fairness |
| I would be very bored by a book about the history of science and technology* | .65 | .037 | 1.02 | O2: Inquisitiveness |
| The first thing that I always do in a new place is to make friends | .65 | .037 | 1.2 | X3: Sociability |
| Having a lot of money is not especially important to me* | .64 | .038 | .97 | H3: Greed Avoidance |
| I find it hard to keep my temper when people insult me* | .64 | .038 | .91 | A4: Patience |
| I have sympathy for people who are less fortunate than I am* | .64 | .038 | .96 | Altruism |
| I rarely, if ever, have trouble sleeping due to stress or anxiety* | .64 | .038 | .98 | E2: Anxiety |
| I would feel afraid if I had to travel in bad weather conditions | .64 | .038 | .78 | E1: Fearfulness |
| I've never really enjoyed looking through an encyclopedia | .64 | .038 | .93 | O2: Inquisitiveness |
| People see me as a hard-hearted person* | .64 | .038 | 1.08 | Altruism |
| When I suffer from a painful experience, I need someone to make me feel comfortable | .64 | .038 | .89 | E3: Dependence |
| When working, I sometimes have difficulties due to being disorganized | .64 | .038 | .98 | C1: Organization |
| I rarely feel anger, even when people treat me quite badly* | .63 | .038 | .81 | A4: Patience |
| I think that I am entitled to more respect than the average person is | .63 | .038 | .9 | H4: Modesty |
| Most people are more upbeat and dynamic than I generally am | .63 | .038 | 1.06 | X4: Liveliness |
| I am an ordinary person who is no better than others* | .62 | .038 | 1.05 | H4: Modesty |
| I feel strong emotions when someone close to me is going away for a long time | .62 | .038 | .87 | E4: Sentimentality |
| I sometimes can't help worrying about little things | .62 | .038 | 1.11 | E2: Anxiety |
| I find it hard to fully forgive someone who has done something mean to me* | .61 | .039 | 1.11 | A1: Forgivingness 8 |
| Sometimes I like to just watch the wind as it blows through the trees* | .61 | .039 | 1.12 | O1: Aesthetic Appreciation 7 |
| I often check my work over repeatedly to find any mistakes* | .6 | .039 | .98 | C3: Perfectionism |
| I plan ahead and organize things, to avoid scrambling at the last minute | .6 | .039 | .79 | C1: Organization |
| I try to give generously to those in need* | .6 | .039 | 1.17 | Altruism |
| I wouldn't spend my time reading a book of poetry* | .6 | .039 | 1.08 | O1: Aesthetic Appreciation |
| When it comes to physical danger, I am very fearful | .6 | .039 | .92 | E1: Fearfulness |
| Even when people make a lot of mistakes, I rarely say anything negative | .59 | .04 | 1.04 | A2: Gentleness |
| I often push myself very hard when trying to achieve a goal | .59 | .04 | .85 | C2: Diligence |
| Most people tend to get angry more quickly than I do | .59 | .04 | .94 | A4: Patience |
| I like people who have unconventional views | .58 | .04 | .65 | O4: Unconventionality |
| I make a lot of mistakes because I don't think before I act | .58 | .04 | 1.05 | C4: Prudence |
| I make decisions based on the feeling of the moment rather than on careful thought | .58 | .04 | .98 | C4: Prudence |
| I prefer to do whatever comes to mind, rather than stick to a plan | .58 | .04 | 1.03 | C4: Prudence |
| If someone has cheated me once, I will always feel suspicious of that person* | .58 | .04 | 1.06 | A1: Forgivingness |
| I rarely express my opinions in group meetings | .57 | .04 | 1.15 | X2: Social Boldness |
| If I want something from a person I dislike, I will act very nicely toward that person in order to get it | .57 | .04 | .93 | H1: Sincerity |
| Whenever I feel worried about something, I want to share my concern with another person* | .57 | .04 | .96 | E3: Dependence |
| I always try to be accurate in my work, even at the expense of time | .56 | .041 | .99 | C3: Perfectionism |
| I think that most people like some aspects of my personality* | .56 | .041 | .83 | X1: Social Self-Esteem |
| It wouldn't bother me to harm someone I didnt like* | .56 | .041 | 1.21 | Altruism |
| I am usually quite flexible in my opinions when people disagree with me | .55 | .041 | .99 | A3: Flexibility |
| I rarely hold a grudge, even against people who have badly wronged me | .55 | .041 | .81 | A1: Forgivingness |
| When working on something, I dont pay much attention to small details | .55 | .041 | .71 | C3: Perfectionism |
| I get very anxious when waiting to hear about an important decision* | .54 | .041 | .92 | E2: Anxiety |
| I tend to be lenient in judging other people | .54 | .042 | .9 | A2: Gentleness |
| I think that paying attention to radical ideas is a waste of time | .54 | .041 | 1.25 | O4: Unconventionality |
| I can handle difficult situations without needing emotional support from anyone else | .53 | .042 | 1.03 | E3: Dependence |
| I find it hard to compromise with people when I really think I'm right* | .53 | .042 | .98 | A3: Flexibility |
| When people tell me that I'm wrong, my first reaction is to argue with them | .52 | .042 | 1.07 | A3: Flexibility |
| I generally accept people’s faults without complaining about them* | .48 | .043 | 1.18 | A2: Gentleness |
| I wouldn't use flattery to get a raise or promotion at work, even if I thought it would succeed | .48 | .043 | .94 | H1: Sincerity |
| I don't allow my impulses to govern my behavior* | .47 | .043 | 1.29 | C4: Prudence |
| I wouldn't want people to treat me as though I were superior to them* | .46 | .044 | 1.14 | H4: Modesty |
| I wouldn't pretend to like someone just to get that person to do favors for me | .39 | .045 | .9 | H1: Sincerity |
| *r*_TT_ = 12-day test-retest reliability. *SE* = standard error for the *r*_TT_ estimate. *SD* = Standard deviation of the item. * indicates items not included in the HEXACO-60. | | | | |
